# Characteristics of incident benzodiazepine recipients among US veterans with posttraumatic stress disorder

**DOI:** 10.1093/ajhp/zxae311

**Published:** 2024-10-29

**Authors:** Komel N Shahid, Katherine Hadlandsmyth, Delaney R Brainerd, Brian C Lund

**Affiliations:** Iowa City Veterans Affairs Health Care System, Iowa City, IA, USA; VA Office of Rural Health, Veterans Rural Health Resource Center–Iowa City (VRHRC-IC), Iowa City Veterans Affairs Health Care System, Iowa City, IA; Department of Anesthesia, Carver College of Medicine, University of Iowa, Iowa City, IA, USA; Iowa City Veterans Affairs Health Care System, Iowa City, IA, USA; VA Office of Rural Health, Veterans Rural Health Resource Center–Iowa City (VRHRC-IC), Iowa City Veterans Affairs Health Care System, Iowa City, IA; Center for Access & Delivery Research and Evaluation, Iowa City Veterans Affairs Health Care System, Iowa City, IA, USA

**Keywords:** benzodiazepine, posttraumatic stress disorder, veteran

## Abstract

**Purpose:**

While benzodiazepine prescribing among veterans with posttraumatic stress disorder (PTSD) declined substantially in the Veterans Health Administration over the past decade, little is known about current incident prescribing. Our objective was therefore to describe patient, provider, facility, and prescribing characteristics among veterans with PTSD who were incident benzodiazepine recipients in 2022 and contrast these to the characteristics for incident recipients in 2012.

**Methods:**

This retrospective observational study included all veterans with PTSD who received an incident benzodiazepine prescription during calendar year 2022 and separately for 2012. The distribution of patient, provider, facility, and benzodiazepine prescribing characteristics was contrasted between years. Stratified subanalyses were conducted by potential non-PTSD benzodiazepine indication, including anxiety and sleep disorders.

**Results:**

A total of 28,310 (6.6%) incident benzodiazepine recipients were identified in 2012, which decreased to 16,776 (1.9%) incident recipients in 2022. The proportion of initial prescriptions written for a days’ supply of 30 or more days decreased from 75.6% to 51.7%, and the proportion who received a second prescription within 6 months decreased from 68.7% to 53.5%. The proportion of patients with diagnoses for potential benzodiazepine indications also increased, including for generalized anxiety disorder (15.1% increase), obsessive compulsive disorders (0.6% increase), panic disorder (6.7% increase), and sleep disorders (22.9% increase).

**Conclusion:**

As incident benzodiazepine prescribing among veterans with PTSD decreased over the past decade, so did the volume of drug dispensed and duration of therapy, while the prevalence of documented prescriptions for non-PTSD indications increased.

**Key Points**

- Among veterans with posttraumatic stress disorder (PTSD), the incidence of benzodiazepine prescribing decreased between calendar years 2012 and 2022, from 6.6% to 1.9%.
- Among incident benzodiazepine recipients with PTSD, documentation of alternative indications, such as anxiety and sleep disorders, increased during this period from approximately 50% to 75%.
- Measures of benzodiazepine prescribing volume decreased among incident recipients during this period.

Of the approximately 18 million US veterans, about 7% have posttraumatic stress disorder (PTSD), which affects female veterans (13 of 100) more than male veterans (6 of 100).^1^ PTSD is characterized by intrusion symptoms (eg, intrusive thoughts, nightmares, dissociative reactions), avoidance of activities or individuals associated with the traumatic event, negative alterations in cognition and mood, and marked alterations in arousal and reactivity (eg, hypervigilance, exaggerated startle response, irritability), which occur at least 1 month following a traumatic event and last for at least 1 month. Collectively, these symptoms may lead to substantial social, occupational, and interpersonal dysfunction.^2^

Current clinical practice guidelines strongly recommend psychotherapy as first-line treatment in the management of PTSD.^3-5^ Selective serotonin reuptake inhibitors and serotonin and norepinephrine reuptake inhibitors are conditionally recommended by guidelines if pharmacological treatment is preferred by the patient. Benzodiazepines are sometimes used to target PTSD symptoms related to anxiety, insomnia, and irritability.^6^ However, joint guidelines from the Department of Veterans Affairs (VA) and Department of Defense (DoD) recommend against benzodiazepine use in this context due to the lack of clear benefit or evidence in adequately powered trials, potential worsening of the overall severity of symptoms, interference with first-line psychotherapies, unfavorable adverse effect profile, and greater risk for abuse or misuse.^4^

Benzodiazepine prescribing in the VA healthcare system has declined substantially, with prevalence rates decreasing from 36.7% to 10.7% among veterans with PTSD between 1999 and 2019.^6,7^ The sharpest declines were observed after 2013 in parallel to several Veterans Health Administration (VHA) initiatives to improve the safety and effectiveness of psychiatric medication prescribing, with a particular emphasis on benzodiazepines.^6^ However, the impact of this more conservative approach to benzodiazepine prescribing has yet to be investigated, particularly with respect to new initiation of benzodiazepines in veterans with PTSD. Therefore, the goal of this study was to contrast the patient, provider, facility, and drug characteristics among veterans with PTSD in the VHA who were recent incident recipients of benzodiazepines (2022) to those of incident recipients before these initiatives (2012). We anticipated that exploring how these characteristics changed over time would yield insight into how patient preferences and prescriber decision-making have changed, specifically in terms of who is considered an appropriate candidate for benzodiazepine initiation and how these drugs are prescribed at initiation.

## Methods

### Data sources.

This retrospective observational study used national administrative data from the VA Corporate Data Warehouse accessed via the VA Informatics and Computing Infrastructure. Mental health diagnoses (PTSD and comorbidities) were identified using International Classification of Diseases (ICD) codes from inpatient and outpatient encounter data. Information concerning dispensed benzodiazepine prescriptions was obtained from outpatient pharmacy data. This study was evaluated by the University of Iowa institutional review board and deemed to not constitute human subjects research.

### Patients.

For the 2022 cohort, all veterans with at least 1 inpatient encounter coded for PTSD (ICD-10 code F43.1x) during 2021 or at least 1 outpatient PTSD-coded encounter during 2021 and a second encounter within the prior 730 days were selected.^6^ The presence of PTSD was established for 2021 to ensure the diagnosis preceded benzodiazepine prescribing during 2022. Identical methods were applied to the 2012 cohort, except that ICD-9 code 309.81 was used to identify PTSD.

For the 2022 cohort of veterans with PTSD, outpatient benzodiazepine prescriptions dispensed during calendar years 2022 and 2021 were used to identify prevalent and incident prescribing. Prevalent benzodiazepine prescribing was defined as any prescription dispensed during 2022, and incident benzodiazepine prescribing was defined as a prescription dispensed during 2022 that was preceded by 365 days with no dispensed benzodiazepine prescriptions.^6^ Preexisting benzodiazepine prescribing was then defined as prevalent benzodiazepine prescribing that was not incident prescribing. Prescriptions indicating a single day of therapy, with a days’ supply value equal to 1, were excluded from calculation of these metrics as these were likely dispensed for presurgical preparation or other situations unrelated to PTSD or other mental health disorders. Parallel methods were applied to the 2012 cohort.

### Characteristics of incident benzodiazepine recipients.

Patient-, provider-, facility-, and drug-level characteristics were assessed among incident benzodiazepine recipients in both the 2022 and 2012 cohorts. Patient characteristics included demographics, rural residence, anxiety disorders (generalized anxiety disorder, panic disorder, obsessive compulsive disorders), other mental health disorders (depressive disorders, alcohol and other substance use disorders), and concurrent sleep disorders.^8^ Concurrent disorders were established by the presence of at least 1 inpatient or outpatient encounter with a corresponding ICD code during the cohort year, using ICD-10 for 2022 and ICD-9 for 2012. The provider issuing the initial benzodiazepine prescription was classified as psychiatry/mental health, primary care, resident (unknown specialty), or other. The primary practice site where the patient received PTSD care was classified as a medical center (VA hospital), urban-located outpatient clinic, or rural-located outpatient clinic. Clinics were classified as urban or rural based on the zip code of their location, mapped to Rural-Urban Commuting Area codes.^9^ Characteristics of the incident benzodiazepine prescription included the specific drug prescribed and the days’ supply value. The initial prescription was classified as potential “as-needed” use if the quantity dispensed was less than the days’ supply value. Subsequent benzodiazepine prescriptions dispensed during the 6 months following the incident prescription were also identified, and 2 metrics were assigned: an indicator for the presence of any subsequent prescription and the total days’ supply dispensed (exclusive of the initial incident prescription).

### Analysis.

Clinical characteristics among incident benzodiazepine recipients were contrasted between the 2022 and 2012 cohorts using descriptive statistics. Additional analyses included stratification by potential benzodiazepine indication of anxiety disorder, sleep disorder, both, or neither of these indications, which were established using ICD-coded encounters.^8^ Inferential statistics were not used in this analysis because complete data for the study population, veterans receiving care for PTSD within the VHA, were observed. The clinical significance of differences in benzodiazepine frequencies across time between the 2012 and 2022 cohorts and between specified diagnostic strata is discussed.

## Results

A total of 556,009 veterans who received care for PTSD in the VHA in 2012 were identified, and this number increased to 929,618 in 2022 (Table 1). The period prevalence of benzodiazepine prescribing decreased from 27.4% in 2012 to 7.6% in 2022, and incident prescribing similarly decreased from 6.6% to 1.9%. Most period-prevalent users in both 2012 and 2022 had received benzodiazepines during the prior year. For example, 53,488 of the 70,264 prevalent recipients in 2022 (76.1%) were preexisting recipients who had been prescribed benzodiazepines during 2021.

**Table 1.** Frequency of Benzodiazepine Prescribing Among Veterans With Posttraumatic Stress Disorder in the Veterans Heath Administration, Calendar Years 2012 and 2022

Prescribing frequency^a^	2012	2022
Period prevalence	152,153/556,009 (27.4)	70,264/929,618 (7.6)
Preexisting	123,843/556,009 (22.3)	53,488/929,618 (5.8)
Incidence^b^	28,310/432,166 (6.6)	16,776/876,130 (1.9)

^a^Data are shown as the number out of the total number (%).

^b^Incidence frequency calculations use a different denominator that excludes preexisting benzodiazepine recipients because these individuals are not at risk for becoming incident recipients.

Although the mean age (52 years) was unchanged from 2012 to 2022, the proportion of veterans aged 65 years or older among incident benzodiazepine recipients increased from 18.4% to 24.5% (Table 2). Women made up a larger proportion of incident recipients in 2022 at 26.0% compared to 11.9% in 2012, whereas the proportion of rural veterans decreased, from 21.4% to 17.0%. The proportion of veterans with concurrent diagnoses for which benzodiazepines may potentially be indicated increased for all such diagnoses from 2012 to 2020. This included absolute increases of 22.9% for sleep disorders, 15.1% for generalized anxiety disorder, 6.7% for panic disorder, and 0.6% for obsessive compulsive disorders. Proportions decreased or remained essentially unchanged for other examined mental health diagnoses.

**Table 2.** Clinical Characteristics of Veterans With Posttraumatic Stress Disorder Dispensed an Incident Benzodiazepine Prescription in the Veterans Health Administration

Clinical characteristic	2012 (n = 28,310)	2022 (n = 16,776)	Absolute change from 2012 to 2022, %
Age, mean (SD), years	52.0 (15.1)	52.0 (14.8)	0
Age ≥65 years, No. (%)	5,197 (18.4)	4,105 (24.5)	+6.1
Sex, No. (%)			
Male	24,943 (88.1)	12,409 (74.0)	–14.1
Female	3,367 (11.9)	4,367 (26.0)	+14.1
Race, No. (%)			
White	21,307 (75.3)	12,305 (73.3)	–2.0
Black or African American	4,658 (16.5)	2,835 (16.9)	+0.4
Other or unknown race	2,345 (8.3)	1,636 (9.8)	+1.5
Hispanic or Latino ethnicity, No. (%)	2,083 (7.4)	1,672 (10.0)	+2.6
Residence, No. (%)			
Urban	22,250 (78.6)	13,919 (83.0)	+4.4
Rural	6,060 (21.4)	2,857 (17.0)	–4.4
Potential benzodiazepine indications, No. (%)			
Sleep disorder	11,968 (42.3)	10,941 (65.2)	+22.9
Generalized anxiety disorder	2,293 (8.1)	3,888 (23.2)	+15.1
Panic disorder	2,293 (8.1)	2,475 (14.8)	+6.7
Obsessive compulsive disorder	456 (1.6)	374 (2.2)	+0.6
Other mental health diagnoses, No. (%)			
Depressive disorder	21,009 (74.2)	12,410 (74.0)	–0.2
Alcohol use disorder	8,137 (28.7)	4,182 (24.9)	–3.8
Other substance use disorder	6,007 (21.2)	3,299 (19.7)	–1.5
Psychotic disorder	3,420 (12.1)	1,028 (6.1)	–6.0

The distribution of practice sites and provider types was largely similar between 2012 and 2022. Of note, approximately 60% of incident benzodiazepine prescriptions in 2022 were written by mental health specialists and approximately 20% were written by primary care providers (Table 3). The most notable changes in distribution of specific benzodiazepines were an increase in lorazepam (from 29.3% to 36.3%) and a decrease in temazepam (from 17.3% to 5.6%). The duration of the initial benzodiazepine prescription also changed substantially from 2012 to 2022, with the proportion of prescriptions with a days’ supply of 30 or more days decreasing from 75.6% to 51.7%. Similarly, the proportion of initial prescriptions likely intended for as-needed use increased from 9.0% to 21.7%. The proportion of veterans who received an additional fill within 6 months of the initial prescription decreased from 68.7% to 53.5%, as did the mean days’ supply dispensed during this period, from 67.6 days to 58.7 days.

**Table 3.** Prescription Characteristics Among Veterans With Posttraumatic Stress Disorder Dispensed an Incident Benzodiazepine Prescription in the Veterans Health Administration

Prescription characteristic	2012 (n = 28,310)	2022 (n = 16,776)
Practice site, No. (%)		
Medical center	15,032 (53.1)	8,173 (48.7)
Urban clinic	10,941 (38.6)	7,429 (44.3)
Rural clinic	2,269 (8.0)	1,162 (6.9)
Unknown	68 (0.2)	12 (0.1)
Provider type, No. (%)		
Psychiatry/mental health	17,316 (61.2)	9,679 (57.7)
Primary care	6,742 (23.8)	3,110 (18.5)
Resident	1,853 (6.5)	964 (5.7)
Unknown or other	2,399 (14.3)	3,023 (18.0)
First benzodiazepine received, No. (%)		
Lorazepam	8,299 (29.3)	6,088 (36.3)
Clonazepam	7,762 (27.4)	4,274 (25.5)
Diazepam	3,317 (11.7)	2,520 (15.0)
Alprazolam	3,430 (12.1)	2,545 (14.6)
Temazepam	4,900 (17.3)	934 (5.6)
Chlordiazepoxide	479 (1.7)	480 (2.9)
Other	123 (0.4)	26 (0.2)
First days’ supply dispensed, mean (SD)	25.9 (11.8)	20.2 (12.2)
≥30-day supply dispensed, No. (%)	21,396 (75.6)	8,680 (51.7)
Potential as-needed use, No. (%)^a^	2,561 (9.0)	3,644 (21.7)
Prescribing in 6 months after initiation		
At least 1 additional fill dispensed, No. (%)	19,459 (68.7)	8,983 (53.5)
Subsequent days’ supply dispensed, mean (SD)	65.8 (67.6)	42.0 (58.7)

^a^Potential as-needed use was defined as the quantity dispensed being less than the days’ supply dispensed, suggesting that use was more infrequent than daily use.

Because of observed increases in the proportion of incident benzodiazepine recipients with anxiety and sleep disorders, a follow-up analysis of benzodiazepine prescribing characteristics was conducted for the 2022 cohort stratifying across these diagnosis groups. The proportion of incident benzodiazepine recipients with neither a sleep disorder nor an anxiety disorder documented decreased from 48.9% in 2012 to 24.5% in 2022 (Table 4). The proportions of recipients with a sleep disorder only, an anxiety disorder only, and both sleep and anxiety disorders all increased. Alprazolam, clonazepam, and lorazepam were more common among patients with an anxiety disorder only, compared to those with only sleep disorders, whereas diazepam and temazepam were more common among those with only a sleep disorder (Table 5). Initial benzodiazepine prescribing characteristics, including the days’ supply dispensed and the proportion with potential as-needed use, did not seem to vary meaningfully among the stratified diagnosis groups. The anxiety disorder only and combined anxiety and sleep disorder groups were the most likely to receive a second benzodiazepine fill within 6 months of initiation, with proportions of 56.7% and 59.4%, respectively, compared to veterans with a sleep disorder only (52.5%) and neither disorder (48.7%).

**Table 4.** Potential Clinical Indications Observed Among Veterans With Posttraumatic Stress Disorder Dispensed an Incident Benzodiazepine Prescription

Potential benzodiazepine indication^a^	2012 (n = 28,310)	2022 (n = 16,776)
Anxiety disorder only	2,498 (8.8)	1,720 (10.2)
Sleep disorder only	9,978 (35.2)	7,115 (42.4)
Anxiety and sleep disorders	1,990 (7.0)	3,826 (22.8)
No sleep or anxiety disorders recorded	13,844 (48.9)	4,115 (24.5)

^a^Data shown as No. (%).

**Table 5.** Benzodiazepine Prescribing Characteristics Among Veterans With Posttraumatic Stress Disorder, Stratified by Potential Clinical Indication, Calendar Year 2022

Prescribing characteristics	Potential benzodiazepine indication			
	Anxiety only (n = 1,720)	Anxiety and sleep (n = 3,826)	Sleep only (n = 7,115)	Neither (n = 4,115)
**At initiation**				
Benzodiazepine, No. (%)				
Alprazolam	308 (17.9)	628 (16.4)	925 (13.0)	593 (14.4)
Chlordiazepoxide	46 (2.7)	126 (3.3)	192 (2.7)	116 (2.8)
Clonazepam	485 (28.2)	1,070 (28.0)	1,773 (24.9)	946 (23.0)
Diazepam	198 (11.5)	436 (11.4)	1,187 (16.7)	699 (17.0)
Lorazepam	640 (37.2)	1,377 (36.0)	2,480 (34.9)	1,591 (38.7)
Other	0 (0)	4 (0.1)	14 (0.2)	8 (0.2)
Temazepam	43 (2.5)	185 (4.8)	544 (7.6)	162 (3.9)
Days’ supply, mean (SD)	20.9 (12.0)	20.7 (12.0)	20.0 (12.2)	20.0 (12.3)
≥30 days’ supply, No. (%)	925 (53.8)	2,025 (52.9)	3,641 (51.2)	2,089 (50.8)
Potential as-needed use, No. (%)^a^	379 (22.0)	883 (23.1)	1,483 (20.8)	899 (21.8)
**Within 6 months after initiation**				
Any refill, No. (%)	976 (56.7)	2,271 (59.4)	3,733 (52.5)	2,003 (48.7)
Dispensed days’ supply, mean (SD)	45.5 (60.0)	48.7 (62.7)	41.1 (58.0)	36.1 (54.7)

^a^Potential as-needed use was defined as the quantity dispensed being less than the days’ supply dispensed, suggesting that use was more infrequent than daily use.

## Discussion

Prevalent benzodiazepine prescribing in the VHA among veterans with PTSD decreased from 27.4% in 2012 to 7.6% in 2022, and incident prescribing decreased from 6.6% to 1.9%. Declining benzodiazepine prescribing in this population has previously been documented, with prevalence rates gradually declining at least since 1999 and a more rapid decline since 2012.^6,7^ Directly comparable data for patients with PTSD outside the VHA are not available, while similarly no data exist specific to incident prescribing. However, several studies have examined benzodiazepine prevalence in general outpatient populations. In the National Ambulatory Medical Care Survey, a nationally representative survey of adult outpatients in the US, the rate of benzodiazepine-associated visits doubled from 3.8% to 7.4% between 2003 and 2015.^10^ Similarly, an analysis of benzodiazepine prescriptions from the US Medical Expenditure Panel Survey found that the total quantity of benzodiazepine prescriptions filled more than tripled from 1996 through 2013.^11^ As an important contrast, an observational study of adults aged 55 years or older across 3 different US healthcare populations reported changes in benzodiazepine prevalence from 2013 to 2017 and found the greatest decreases in the VHA (from 5.7% to 3.0%), compared to a decrease from 10.4% to 9.3% in fee-for-service Medicare users and 6.6% to 6.5% in commercially insured individuals.^12^ More recent data from employer-sponsored commercial health insurance beneficiaries in the US have noted a gradual decrease in Schedule IV depressants (benzodiazepines and Z-drug hypnotics) from a peak prevalence of approximately 4.7% in 2013 to 3.2% in 2019.^13^ Declines in benzodiazepine prescribing in the VHA, and specifically among veterans with PTSD, have followed a unique trajectory that is likely attributable to a combination of directed initiatives.^6,12^ Importantly, these changes have not been accompanied by compensatory increases in prescribing of alternative sedatives or hypnotics, such as Z-drug hypnotics, trazodone, and low-dose quetiapine.^6^

Against the backdrop of declining benzodiazepine prescribing in veterans with PTSD, the focus of this analysis was to examine how patient and prescribing characteristics among new recipients have changed over the past decade, with the hope of gaining insight into shifts in patient preferences and provider decision-making that have occurred during this period of relatively rapid change. Substantial changes have occurred in the past 10 years in prescribing characteristics among incident benzodiazepine recipients. Fewer days’ supply was dispensed on the initial fill (decreasing from 25.9 days in 2012 to 20.2 days in 2022) and subsequent fills within 6 months (decreasing from 65.8 days to 42.0 days). These findings indicate not only that benzodiazepines are less likely to be initiated, but also that the volume and duration of therapy, when prescribed, have decreased. These findings for initial benzodiazepine prescriptions are consistent with prior research demonstrating decreasing days’ supply dispensed among prevalent recipients.^6^ We further observed an increase in the proportion of initial benzodiazepine prescriptions likely intended to be taken on an as-needed basis, rather than for regular daily use. These combined findings may imply that providers have become increasingly cognizant of minimizing benzodiazepine exposure in this patient population; future work is needed to explore this possibility. Finally, the proportion of veterans 65 years or older increased over time among incident benzodiazepine recipients, from 18.4% to 24.5%. Similar findings in the VHA have previously been reported and warrant further investigation.^6^

With respect to patient characteristics, the most notable change was that incident benzodiazepine recipients in 2022 were more likely to have been diagnosed with comorbid anxiety and sleep disorders relative to 2012. The proportion of veterans without a documented comorbid sleep or anxiety disorder decreased from 48.9% to 24.5% during this period. These findings may suggest that providers have become less likely to prescribe benzodiazepines for core symptoms of PTSD and more likely to target comorbid indications such as anxiety or insomnia. However, an alternative explanation may be that providers have become more likely to document a formal diagnosis instead of managing symptoms in the absence of a diagnosis. Unfortunately, a limitation of this research was that we could not directly ascertain for which indications or symptoms a medication was prescribed using administrative data. In attempting to integrate patient and prescribing characteristics in the 2022 cohort, we contrasted characteristics for incident benzodiazepine prescribing across patient groups with and without sleep and anxiety disorders. While we expected markedly fewer days’ supply dispensed and lower refill rates among patients with sleep disorders relative to those with anxiety disorders, we failed to observe any consistent and substantial differences in benzodiazepine prescribing characteristics between these groups. These findings suggest that tailoring care to specific comorbid indications, where short-term, intermittent use may be more appropriate for sleep disorders while longer-term, scheduled use may be more appropriate for anxiety disorders, may not be driving incident benzodiazepine prescribing as much as anticipated.

This analysis was not without limitations. First, some veterans may have received a benzodiazepine prescription from a non-VA source, which we were unable to identify in our data. Specifically, it is unclear whether more conservative benzodiazepine prescribing practices at the VHA have shifted prescribing to non-VA providers. Unfortunately, there is limited data examining this question. However, among older adults who were dually enrolled in the VHA and Medicare, the prevalence of benzodiazepine dispensing by the VHA decreased from 5.2% to 3.1% between 2013 and 2017, with no observed increase in benzodiazepine dispensing through Medicare Part D during this period.^14^ Additionally, these results may not be generalizable to non-VA settings due to demographic differences in the distribution of age, sex, and race among veterans relative to the civilian population and the specific availability and implementation of VA/DoD PTSD clinical practice guidelines within the VA healthcare system. Finally, it is possible that as-needed benzodiazepine prescribing may have been underestimated in this study; some providers may write full prescriptions for as-needed use, which we were unable to determine in this administrative dataset.

## Conclusion

Several characteristics of incident benzodiazepine recipients changed over the period from 2012 to 2022 among veterans with PTSD. Notably, alternative non-PTSD indications were more commonly documented in 2022 and several aspects of prescribing volume decreased, including initial days’ supply prescribed and total duration of therapy. Efforts to curtail benzodiazepine prescribing in veterans with PTSD have produced additional positive prescribing changes among incident recipients that are consistent with minimizing unnecessary exposure and selective prescribing for other indications. While incident benzodiazepine prescribing continues to be minimized, in terms of both frequency and volume, it is important for providers to assess the ongoing appropriateness of benzodiazepine prescribing in veterans with PTSD.

## Data Availability

The data underlying this article cannot be shared publicly because they are Veterans Health Administration data.
